# Malarial Hæmaturia

**Published:** 1885-09

**Authors:** H. McHatton

**Affiliations:** Macon, Ga.


### CLINICAL HISTORY OF A CASE OF (SO-CALLED) MALARIAL HÆMATURIA.

BY H. MCHATTON, MACON, GA.

Reported for THE ATLANTA MEDICAL AND SURGICAL JOURNAL.

Mr. X., white, 35 years old, has an old stricture that has given him considerable trouble; been having chills for the past two months; has been using a moderate amount of quinine and large doses of calomel (xv to xx grs); had been having a diarrhœa for 24 hours, for which he had taken about xxx grs. of calomel. At noon he had a violent chill, during which he began to pass dark colored urine.

Saw him at 3 p. m.—Pulse 100; temperature 101. Passing urine in large quantities about every half hour. Gave morph. sul. gr. 1-6; quiniæ sulph., gr. xx.

8 p. m.—Temperature 102. Vomited medicine. Quiniæ sul. gr. x. Repeat in two hours.

2d day, 9 a. m.—Pulse 80; temperature 101. Complete jaundice. Nausea and vomiting all night. Morph. sul. gr. 1-6, hypodermically. Quin. sul. gr. x.

11:45 a. m.—Temperature 100; pulse 80. Nausea but no vomiting. Pulse irregular but very weak, as is also the first cardiac sound. Quin. sul. gr. x. Tr. digitalis gtts. x.

12 noon.—Pulse 80; temperature 102. Vomited medicine. Quin. sul. gr. v. Given hypodermically.

4 p. m.—Pulse 80; temperature 101. No vomiting. Quin. sul. gr. v. Hypodermically. Spts. ammon aromat ʒss. q. 3.h. Passes urine copiously about once an hour.

7:30 p. m.—Pulse 84; temperature 102. Urine same. No passage from bowels since first day. Drank a little soup and tea during the evening. Morph. sul. gr. 1-6. Quin. sul. gr. v. Hypodermically. Continue ammonia.

11 p. m.—Temperature 100 1-2; pulse 80; respiration 20. Continuous nausea and vomiting. Large passage from bowels—liquid and very dark. Morph. sul. gr. 1-4. Hypodermically.

3d day, 8 a. m.—Pulse 80; temperature 99. Has retained nothing since 3 a. m. No change in urine. Slept very little—only short naps. Mustard plaster to stomach.

11 a. m.—Pulse 88; temperature 98 1-2. Retains nothing. Ordered enema mixture of yolk of one egg, whisky and milk each ʒiii, morph. sul. gr. 1-4. Gave ℥ss of the mixture. Repeat in ounce doses at 3 and 6 o'clock. Champagne ʒi q. 1-2. h., if retained.

9 p. m.—Pulse 88; temperature 98 1-2. Constant retching, though enema retained. Only ℥iv urine since 11 a. m—not so highly colored. Morph. gr. 1-4, and whisky ℥ss. Hypodermically. Repeat enema 9. 4. h. Nothing by stomach.

4th day, 9 a. m.—Pulse 80; temperature 98 1-2. No vomiting from 9 to 3 o'clock—vomiting ever since. Three enemas retained 1-6 gr. morph. in second; none in other two. Has taken nothing during the night by the mouth. Urine ℥viii, clear.

10:45 a. m.—Pulse 90; temperature 99. Five gr. quin. in enema mixture. Morph. sul. gr. 1-4. Hypodermically. Chloroform gtt. i, aq. calcis ʒi every hour.

4 1-2 p. m.—Saw in consultation with Drs Holt and Williams, who continued in attendance till conclusion of the case.

Pulse 110. Urine ℥iii. Quin. sul. grs. iii. Morph. sul. gr.

1-6. Whisky ʒss. Hypodermically. ʒi doses of milk and lime water *aa* if retained. 4x4 epigastric blister. Continue enema q. 3. h.

8:50 p. m.—Pulse 120. Nausea and retching continuous. No vomiting. Blister has not drawn. Whisky ʒss. Hypodermically. Enema—milk and lime water—continued.

5th day, 8:30 a. m.—Pulse 120; temperature 98.6. Axillary (previous temperatures taken in the mouth.) Nausea and retching all night. No vomiting. Urine ℥iv. Slept fairly well. Blister drew a dark colored liquid. Perfectly conscious. Tr. digitalis gtt x.

11:30 a. m.—Pulse 110; temperature 98.6. Neutral mixture potass. bitart. ʒii q. h.

4 p. m.—Pulse 120; temperature 99. Slept one hour. Potass. mixture not retained a moment. Enemas retained. Has taken a little champagne. Tr. digitalis gtts x.

9:30 p. m.—Pulse 125; temperature 98.8. Retains nothing but enemas. Urine ℥iii. Considerable hepatude, but mind clear when aroused. Continue stimulants as well as possible.

6th day, 7:30 a. m.—Pulse 125; temperature 98.9. Constant hiccough. Nausea, but no vomiting. Bowels moved once. Enema retained the rest of the time. Amyl nitrate for the hiccough—stopped immediately. Continue stimulants.

12 noon.—Pulse 135. Hiccough returned fifteen minutes after amyl was used and continues yet. Urine ℥iv. Stimulants as can be borne, and hot applications to stomach.

3:30 p. m.—Temperature 98.8; pulse 122. No vomiting. small amount of stimulants and enemas taken.

9 p. m.—Pulse 115; temperature 99. Hiccough unchanged. Continue same treatment.

7th day, 9:30 a. m.—Pulse 110. Hiccough all night. Added beef tea to enema instead of egg.

3 p. m. Pulse 100; temperature 98.5

Died at 10 p. m., conscious up to the last.

The urine in this case was at first a brownish black and loaded with the chemical constituents of the blood. Several of the specimens were under the microscope within ten minutes after they passed, but there were none of the anatomical elements except an occasional corpuscle, say at most three or four to the slide, which could be accounted for by his previous urethral condition. In many of the specimens there were none at all, but these same specimens gave hæmin crystals in abundance. The condition of the urine improved continuously after the third day, and before his death I could get no evidences of hæmoglobinuria.

The vomited matters never gave any of the gross evidences of blood, so they were not examined chemically. I regret I did not examine the passage from the bowels on the second day, but it was thrown away without my knowledge.

The jaundice was the most intense I ever saw and came on in its full intensity the first night, remaining about the same until death. Whereas, the pulse and temperature in the early part of the disease were not high or fast enough to give alarm, there were symptoms that clearly showed danger; the pulse was full, but obliterated by very slight pressure; first heart sound weak and valvular. Respiration often sighing, and a most anxious countenance. From the first the patient had no hopes of recovery. If I had the case to treat again I would begin the hypodermics of quinia and rectal alimentation earlier, leaving the stomach as quiet as possible. At the same time, I fear that it would not have altered the final result.

My impression is that, during the initial chill, the destruction of blood corpuscles was so great as to render the continuance of life impossible. If not, what was the cause of death? It certainly was not hyperpyrexia. He could not have starved to death with the amount of rectal alimentation that he got and retained. I think that uræmic can be eliminated. There were no convulsions; the mind was clear. There was no trouble with the sight, the only Uræmic symptom being the persistent nausea which came on early in the disease. The repeated examinations of the urine gave no signs of acute or chronic nephritis.

